# Heart rate turbulence in type 2 diabetes mellitus with chronic kidney disease

**DOI:** 10.1371/journal.pone.0319116

**Published:** 2025-07-15

**Authors:** Huimin Zhu, You-Fan Peng, Huiping Gu, Xingjing Liu

**Affiliations:** 1 Department of Electrophysiology, The Affiliated Huaian No.1 People’s Hospital of Nanjing Medical University, Huaian, China; 2 Department of Respiratory and Critical Care Medicine, Affiliated Hospital of Youjiang Medical University for Nationalities, Baise, China; 3 Life Science and Clinical Medicine Research Center, Affiliated Hospital of Youjiang Medical University for Nationalities, Baise, China; 4 Department of Endocrinology, The Affiliated Huaian No.1 People’s Hospital of Nanjing Medical University, Huaian, China; Ospedale San Pietro Fatebenefratelli, ITALY

## Abstract

**Objective:**

Heart rate turbulence (HRT), a test quantified by turbulence onset (TO) and turbulence slope (TS), has been used to assess cardiac autonomic neuropathy (CAN). CAN is a complication of chronic kidney disease (CKD) in patients with type 2 diabetes mellitus (T2DM). Thus, this study aimed to assess the association between HRT parameters and CKD in T2DM patients.

**Methods:**

This retrospective study included 322 patients with T2DM, grouping them into those with and those without CKD; the data collected from the patients with T2DM were analysed. T2DM was diagnosed according to American Diabetes Association criteria. CKD diagnoses were confirmed according to KDIGO Clinical Practice Guidelines.

**Results:**

For HRT parameters, T2DM patients with CKD exhibited a significantly higher turbulence onset (TO) (−0.25 [−1.02, 0.23] vs −0.64 [−1.93 0.10], *p* = 0.034) and lower turbulence slope (TS) (2.49 [1.27, 4.00] vs 6.20 [2.55, 8.80], *p* < 0.001) than those without CKD. TO was significantly negatively correlated with estimated glomerular filtration rate (eGFR) (r = −0.184, *P* = 0.001), whereas TS was significantly positively correlated with eGFR in patients with T2DM (r = 0.203, *P* < 0.001). Multivariable logistic regression analysis revealed that higher TS, but not for TO, was independently associated with CKD in patients with T2DM(OR=0.885, 95%CI:0.8400.932, *P* < 0.001).

**Conclusion:**

Higher TS, an HRT parameter reflecting heart rate deceleration following ventricular premature beat, was independently associated with CKD in T2DM patients. This study suggests that CKD may change heart rate deceleration following ventricular premature beat in T2DM patients, which may be helpful in improving CAN assessment by HRT in patients with CKD.

## 1. Introduction

The increasing morbidity of type 2 diabetes mellitus (T2DM) is a substantial global health challenge [1]. The main chronic complications of T2DM are involved in nephropathy, retinopathy, and neuropathy [2–4]. Thereinto, Diabetic nephropathy is the leading cause of chronic kidney disease (CKD) among patients with T2DM [5]. Patients with T2DM who suffer from CKD exhibit significant susceptibility to the occurrence and development of cardiovascular disease [6]. While CKD is a significant independent risk factor for adverse cardiovascular outcomes, cardiovascular disease in T2DM patients with CKD is attributable to multiple risk factors [7]. Therefore, the clinical management of cardiovascular risks in T2DM patients with CKD requires greater attention.

Heart rate turbulence (HRT) is quantified by turbulence onset (TO), which reflects an initial acceleration of heart rate following ventricular premature beat (VPB) and turbulence slope (TS) which describes subsequent heart rate deceleration [8]. The HRT parameters have been recognized as reliable indicators of baroreceptor sensitivity, with its biphasic response primarily mediated by the vagus nerve [9]. The HRT assessment is considered applicable to risk stratification following acute myocardial infarction as well as to the prediction and monitoring of the progression of heart failure and several other pathological diseases [10–12]. Patients with myocardial infarction, particularly those with diabetes, exhibit abnormal HRT and have an elevated risk of life-threatening ventricular arrhythmias and cardiac mortality [10,13]. The HRT parameters can serve as effective predictors of all-cause mortality risk in both diabetic and non-diabetic myocardial infarction patients [13]. Significant improvements in HRT parameters both TO and TS have been observed in T2DM patients who receive insulin therapy for intensive glycemic control [14]. Cardiac autonomic neuropathy (CAN) is a common chronic complication characterised by dysfunction of the autonomic nervous in the cardiovascular system [15]. It has been demonstrated to be associated with postural hypotension, asymptomatic myocardial ischemia and myocardial infarction [16]. Not only is CAN a prevalent complication of diabetes, but it has also been recognized as a significant risk factor for the development and progression of CKD [6,17]. Indeed, abnormal HRT has been shown to be prevalent in patients with CKD undergoing hemodialysis [18]. Additionally, abnormal CAN has been closely associated with CKD, and has been shown to independently predict a decline in estimated glomerular filtration rate (eGFR) in patients with T2DM [19]. It has been reported that HRT is useful in monitoring changes in the CAN of T2DM patients [20]. Thus, the aim of this study was to investigate the possible associations between HRT parameters and CKD in patients with T2DM.

## 2. Methods

### 2.1 Patients

This retrospective study included 377 patients with T2DM who visited the Affiliated Huaian No.1 People’s Hospital of Nanjing Medical University between 1^st^ December 2022 and 29^th^ March 2024. T2DM diagnosis was based on American Diabetes Association criteria [21]. CKD was diagnosed in accordance with the KDIGO Clinical Practice Guidelines [22]. T2DM patients who experienced atrial fibrillation, bundle branch block, hemodynamically unstable valvular heart disease, congenital heart abnormalities, past myocardial infarction, recurrent premature ventricular contractions, or who had implanted cardiac rhythm management devices or cancer were excluded from the study, along with those who were pregnant or using cardioactive medication. In addition, T2DM patients with missing data were excluded from the analysis. Before this study started initiated in 3^rd^ July 2024, ethical approval was granted by the Ethics Committee of the Affiliated Huaian No.1 People’s Hospital of Nanjing Medical University, and this study adhered to the Declaration of Helsinki. Given that it was a retrospective study, the requirement for informed consent was waived.

### 2.2. Data collection

The following medical data were collected from electronic medical records: gender, age, height, weight, medical history, laboratory test results including hemoglobin Alc (HbA1c), triglyceride (TG), total cholesterol (TC), high-density lipoprotein cholesterol (HDL-C), low-density lipoprotein cholesterol (LDL-C), and creatinine (Cr), and 24-hour Holter records. The estimated glomerular filtration rate (eGFR) was calculated with adjustment of Asians [23].

### 2.3. Definition

Body mass index (BMI) was calculated: BMI = body weight (kg)/ height (m^2^). A 24-hour Holter recording was performed on each subject to measure the HRT components (TO and TS).

Turbulence onset is calculated as:


$$TO=\frac{\left(RR_{1}+RR_{2}\right)-\left(RR_{-2}+RR_{-1}\right)}{\left(RR_{-2}+RR_{-1}\right)}\times 100[\begin{array}{c} \% \end{array}]$$

RR_-2_ and RR_-1_ represent, respectively, the two sinus R-R intervals VPBs, while RR_1_ and RR_2_ represent the two consecutive sinus R-R intervals following the compensatory pause of VPBs. TS is defined as the maximum positive regression slope derived from any set of five consecutive sinus rhythm R-R intervals within the first fifteen sinus rhythm R-R intervals following a VPB [10]. Holter ECG was performed on a three-channel digitised recorder (GE Medical Systems, USA).

### 2.4 Statistical analysis

The Shapiro-Wilk test was used to test the normality of continuous variables. Categorical variables are described as frequency and percentage. Continuous variables with non-normal distribution are described as median and interquartile range. The Chi-square test was used to determine significant differences in categorical variables, and the Mann-Whitney U test was used to determine significant differences in continuous variables. Spearman’s correlation coefficient was applied to analyse the correlations. Multivariable logistic regression analysis was used to determine the independent factors associated with CKD in patients with T2DM. A p-value <0.05 was considered statistically significant. PASS 2023 was employed to determine the required sample size (NCSS Corporation, Kaysville, Utah, USA). The data analyses were performed using SPSS version 25.0 (IBM Corporation, Armonk, NY, USA).

## 3. Results

### 3.1. The characteristics of T2DM patients with and those without CKD

The characteristics of T2DM patients with and those without CKD are summarized in Table 1. TO (−0.25 [−1.02, 0.23] vs −0.64 [−1.93 0.10] *p* = 0.034) was significantly increased and TS (2.49 [1.27, 4.00] vs 6.20 [2.55, 8.80], *p* < 0.001) was significantly decreased in T2DM patients with CKD compared to those without CKD. Moreover, serum HDL-C levels were significantly lower in T2DM patients with CKD than in those without it (*p* = 0.050). There were no significant differences between the groups regarding age(*p* = 0.525), sex(*p* = 0.976), BMI(*p* = 0.724), HbA1c(*p* = 0.630), TC(*p* = 0.377), TG(*p* = 0.189), LDL-C(*p* = 0.102), hypertension history(*p* = 0.608), and cardiovascular disease history (*p* = 0.550).

**Table 1:** The characteristics of T2DM patients with and those without CKD.

	T2DM with CKD (N = 150)	T2DM without CKD (N = 172)	*p* value
Age (years)	66(55.75,75)	67(61,74)	0.525
Sex (M/F)	77/73	88/84	0.976
Hypertension history (%)	106(70%)	117 (68.02%)	0.608
Cardiovascular disease (%)	80 (53.33%)	86(50%)	0.550
Body mass index (kg/m^2^)	24.78(22.86,28.34)	25.10(23.19,27.60)	0.724
Hemoglobin Alc (%)	8(7,9.50)	8.10(7,9.25)	0.630
Total cholesterol (mmol/L)	3.63(2.86,4.74)	3.41(2.94,4.35)	0.377
Triglyceride (mmol/L)	1.56(1.08,2.26)	1.46(0.99,2.07)	0.189
High-density lipoprotein cholesterol(mmol/L)	1(0.81,1.20)	1.03(0.84,1.30)	0.050
Low-density lipoprotein cholesterol (mmol/L)	1.88(1.46,2.75)	1.92(1.32,2.37)	0.102
Turbulence onset	−0.25 (−1.02, 0.23)	−0.64 (−1.93, 0.10)	0.034
Turbulence slope (%)	2.49 (1.27, 4.00)	6.20 (2.55, 8.80)	<0.001

### 3.2. The correlation assessment between HRT parameters and renal function in patients with T2DM

The results of correlation analysis in patients with T2DM are summarized in Table 2. TO was significantly negatively correlated with eGFR (*r* = −0.184, *P* = 0.001), and TS was significantly positively correlated with eGFR (*r* = 0.203, *P* < 0.001). In addition, TO was significantly positively correlated with age (*r* = 0.160, *p* = 0.004), and TS was significantly negatively correlated with age (*r* = −0.152, *p* = 0.006), BMI (*r* = −0.116, *p* = 0.038), HbA1c (*r* = −0.118, *p* = 0.034), TC (*r* = −0.167, *p* = 0.003), and LDL-C (*r* = −0.145, *p* = 0.009).

**Table 2:** The correlation between HRT parameters and continuous variables in patients with T2DM.

	TO		TS	
	r	*p* value	r	*p* value
Age	0.160	0.004	−0.152	0.006
Body mass index	−0.081	0.145	−0.116	0.038
Hemoglobin Alc	−0.048	0.389	−0.118	0.034
Total cholesterol	−0.010	0.860	−0.167	0.003
Triglyceride	−0.079	0.158	−0.107	0.055
High-density lipoprotein cholesterol	−0.052	0.352	0.061	0.279
Low-density lipoprotein cholesterol	0.011	0.849	−0.145	0.009
Estimated glomerular filtration rate	−0.184	0.001	0.203	<0.001

### 3.3. The association between HRT parameters and CKD by multivariable logistic regression analysis in patients with T2DM

The results of multivariable logistic regression analysis in patients with T2DM are summarized in Table 3 and 4. After age, sex, hypertension history, cardiovascular disease history, BMI, HbA1C, TC, TG, LDL-C, and HDL-C were adjusted in each of the two models, the multivariable logistic regression analysis showed that higher TS (OR=0.885, 95%CI:0.840–0.932, *p* < 0.001), but not for TO (OR=1.006, 95%CI:0.924–1.095, *p* = 0.893), was independently associated with CKD, and that lower HDL-C (OR=0.297, 95%CI:0.116–0.761, *p* = 0.011; OR=0.222, 95%CI:0.084–0.585, *p *= 0.002) remained an independent association with CKD in patients with T2DM.

**Table 3:** The multivariable logistic regression analysis between TS and CKD in patients with T2DM.

	OR (95%CI)	*p* value
Age	0.988(0.964,1.012)	0.310
Sex	0.942(0.578,1.536)	0.811
Hypertension History	0.908(0.505,1.631)	0.747
Cardiovascular Disease	0.931(0.541,1.603)	0.796
Body mass index	0.966(0.913,1.021)	0.219
Hemoglobin Alc	0.898(0.775,1.041)	0.155
Total cholesterol	1.286 (0.744,2.223)	0.367
Triglyceride	0.755 (0.541,1.055)	0.100
Low-density lipoprotein cholesterol	1.194(0.666,2.140)	0.553
High-density lipoprotein cholesterol	0.297 (0.116,0.761)	0.011
Turbulence slope	0.885(0.840,0.932)	<0.001

**Table 4:** The multivariable logistic regression analysis between TO and CKD in patients with T2DM.

	OR (95%CI)	*p* value
Age	0.995(0.972,1.019)	0.677
Sex	1.010(0.633,1.613)	0.965
Hypertension History	0.792(0.454,1.381)	0.410
Cardiovascular Disease	0.804(0.482,1.340)	0.402
Body mass index	0.984(0.931,1.039)	0.561
Hemoglobin Alc	0.921(0.802,1.058)	0.244
Total cholesterol	1.395 (0.798,2.437)	0.243
Triglyceride	0.749(0.533,1.051)	0.095
Low-density lipoprotein cholesterol	1.208(0.670,2.179)	0.530
High-density lipoprotein cholesterol	0.222 (0.084,0.585)	0.002
Turbulence Onset	1.006(0.924,1.095)	0.893

## 4. Discussion

This study investigated the associations between HRT and CKD in patients with T2DM and found that higher TS, rather than TO, was independently associated with CKD in these individuals. HRT reflects transient hemodynamic disturbances induced by VPBs; these disturbances are characterised by changes in the cycle length of sinus rhythm following VPB, mediated through baroreflex mechanisms [10]. Specifically, the temporary decrease in blood pressure caused by VPB activates baroreceptors, which suppress vagal nerve activity and thereby accelerate heart rate [10]. This constitutes the primary mechanism underlying TO [24]. Simultaneously, the drop in blood pressure triggers activation of the sympathetic nervous system, with enhanced sympathetic responses leading to a gradual increase in systolic blood pressure and vascular resistance [25]. To maintain autonomic balance between the sympathetic and parasympathetic nervous systems, vagal activity is subsequently re-enhanced, a process quantitatively assessed by the TS [25]. Therefore, normal HRT depends on the intricate interactions between the vagus nerve and sympathetic nervous system. Any cause of abnormality to these two systems can cause abnormal HRT.

Several studies have demonstrated a significant correlation between CAN and CKD in diabetic patients [26,27]. It is widely recognized that sympathetic nerve function exerts a more substantial influence on renal function compared to the vagus nervous system, as the renal glomeruli and renal tubules are predominantly innervated by sympathetic nerves [28]. In diabetic patients with CAN, the deterioration of vagus function typically precedes the development of sympathetic neuropathy and may subsequently induce a relative increase in sympathetic activity [29]. Tang et al [30] found that CAN was a strong predictor of declined kidney function in patients with either Type 1 diabetes mellitus or T2DM. Alterations in sympathetic nerve activity and imbalances of the autonomic nervous system can disrupt the homeostasis of the renal internal environment, thereby expediting the progression of renal insufficiency [27]. This further highlights the autonomic nervous system’s critical role in HRT. In addition, HRT is also significantly associated with baroreflex sensitivity, and its origin is thought to be an autonomic response to blood pressure disturbance following premature ventricular depolarization [9]. CAN may attenuate nocturnal blood pressure, potentially rendering the kidney more susceptible to overall burden [31]. It has been demonstrated that HRT values deteriorate more markedly in hemodialysis patients with hypotension than in those with normal blood pressure during non-dialysis periods [32]. Valensi et al [27] suggested that the loss of neural regulation of renal haemodynamics might render the kidney more susceptible to the haemodynamic impact of systolic blood pressure. Furthermore, Rubinger et al [33] revealed that in the long term, blood pressure and baroreflex function were restored to near normal range in kidney transplant patients. Based on the mechanisms, the phenomenon of weakened HRT can be observed in T2DM patients with CKD.

Balcioglu et al [20] found that T2DM patients with CAN had lower TS values, but similar TO values, compared with those without CAN. This finding may align with our study suggests that TS rather than TO, constitutes an independent risk factor for T2DM with CKD. A plausible explanation for this disparity is that CAN, itself represents a condition wherein the sympathetic nervous system is relatively dominant [20]. TO merely reflects the initial acceleration following a VPB, indicating the transient activation and instantaneous response of the sympathetic nervous system [10]. By contrast, TS can reflect the vagus nerve’s restorative activity and overall regulatory capacity by demonstrating slope trends across multiple RR intervals, which might explain why it serves as an independent risk factor for CAN [10]. The present study confirmed a significant correlation between HRT parameters and age in patients with T2DM, consistent with prior investigations [34]. The study also found that lower HDL-C levels were significantly associated with CKD in patients with T2DM. Reduced HDL-C levels have been associated with the progression of kidney disease, particularly in patients undergoing haemodialysis [35]. Hypertension has been regarded as an independent risk factor for CKD in patients with T2DM [36]. However, the present study did not observe the association between hypertension history and CKD in patients with T2DM; hypertension duration and medication use were considered the possible main factors.

This study should consider several limitations. First, it is a retrospective analysis, hence, other potential factors may have affected the observed results. Second, due to the study’s cross-sectional design, its findings cannot assess whether HRT is an effective predictor for declined kidney function in patients with T2DM. Third, the study was limited to patients with T2DM, so its findings may not apply to other populations.

## 5. Conclusion

The present study demonstrated that higher TS, an HRT parameter quantifying deceleration of heart rate following VPB, exhibited an independent association with CKD in patients with T2DM. This suggests that CKD exerts a substantial influence on the deceleration of heart rate after premature beat in patients with T2DM, thus potentially enhancing the clinical utility of HRT assessment for detecting CAN in T2DM patients with CKD.
